# Scientific and Regulatory Perspectives on Chemical Risk Assessment of Pesticides in the European Union

**DOI:** 10.3390/jox15050173

**Published:** 2025-10-21

**Authors:** Fabio Buonsenso

**Affiliations:** Department of Chemistry “Ugo Schiff”, University of Florence, Via della Lastruccia 3-13, Sesto Fiorentino, 50019 Florence, Italy; fabio.buonsenso@unifi.it

**Keywords:** climate change scenarios, CLP regulation, REACH, endocrine disruptors, immunotoxics, EU regulation, cumulative exposure, human health and environmental protection, plant protection

## Abstract

People are exposed to pesticides daily through food, drinking water, and the environment, both in urban and rural settings. These chemicals, while offering economic and agricultural benefits through pest control and increased productivity, may pose a growing risk to human health and ecosystem biodiversity. While the European regulatory framework offers a robust foundation for risk assessment, significant limitations persist, especially in addressing cumulative exposure, low-dose effects, and chemical mixtures. This review focuses on selected scientific and regulatory challenges by reviewing recent *European Food Safety Authority* (EFSA) conclusions, *Organization for Economic Co-operation and Development* (OECD) test guidelines updates, and current European legislative approaches. Particular attention is given to the regulation of endocrine-disrupting and reprotoxic substances, highlighting progress and remaining gaps in implementation. A brief mention will also be made of immuno-toxic substances, for which no specific hazard class has yet been established. Building on official reports and peer-reviewed literature, this review provides a structured evaluation of the scientific and regulatory landscape, including underexplored issues like the transition to animal-free toxicology and integration of biomonitoring with health data. The goal is to propose realistic, evidence-based improvements to current frameworks using integrated, interdisciplinary approaches that connect toxicology, policy, and implementation science. A shift to a holistic, systems-based, and precautionary paradigm is vital to address emerging challenges and ensure strong protection of health and environment, as well as supporting the needs of the agricultural sector.

## 1. Introduction

**Figure 1:**
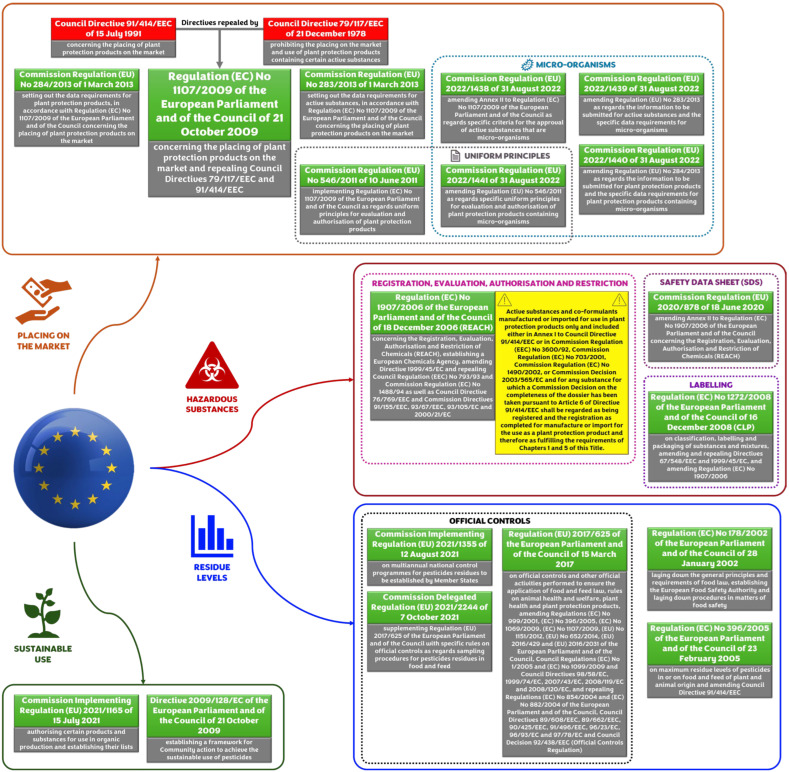
Overview of European legislation on plant protection products. Green box: legislation in force; red box: legislation no longer in force.

Plant protection products (PPPs), including herbicides, fungicides, and insecticides, play a crucial role for sustaining agricultural productivity and ensuring global food security [1]. However, their widespread and prolonged use continues to raise significant concerns about their long-term effects on human health and ecosystems, in particular in the context of chronic, aggregate, and cumulative exposure [2,3]. Risk assessment associated with the use of PPPs represents an extremely complex challenge, reflecting the variety and dynamic nature of the scenarios in which these risks arise. It is essential to adopt a rigorous scientific approach, based on sustainable and reliable procedures, capable of ensuring the highest possible level of safety, in light of current knowledge. Consequently, is also essential an exhaustive regulatory control, aimed at protecting public health and the environment [4]. This need motivates increasing attention and a continuous investment of resources to ensure a constant evolution of the risk assessment and applicable mitigation measures. In the European Union (EU), pesticide regulation (Figure 1) is primarily governed by Regulation (EC) No 1107/2009, together with broader legislative instruments such as the *Registration*, *Evaluation*, *Authorisation and Restriction of Chemicals* (REACH) regulation and the *Classification*, *Labelling and Packaging* (CLP) regulation [5,6,7,8]. REACH and CLP provide standardized hazard classification, labeling, and exposure assessment criteria that complement the specific requirements of pesticide regulation.

Although this framework is designed to be comprehensive and well-structured, scientific and operational limitations continue to hinder its effective implementation. These include the limited integration of realistic exposure scenarios, insufficient attention to mixture toxicity, and regulatory uncertainty regarding low-dose effects and non-monotonic responses [9,10]. Despite some recent progress, the evaluation and management of endocrine-disrupting and reprotoxic substances remain fragmented and unevenly implemented across Member States [11,12].

Building on this historical and regulatory context, the focus now turns to emerging challenges posed by these substances. The persistent gaps in their assessment and management highlight the need for integrated, multidimensional approaches that account for cumulative exposure, low-dose effects, and environmental persistence. From an operational standpoint, regulatory flexibility tools, such as emergency authorizations for non-approved active substances, are frequently employed in EU countries. In some cases, these authorizations are issued repeatedly or extended beyond standard timeframes. This trend reflects the practical challenges of reconciling regulatory objectives with the demands of agricultural production [13,14]. Similarly, notable variability in pesticide use can be observed across different crops and Member States, influenced by national policies, enforcement approaches, and specific agronomic conditions, and the occurrence of so-called “minor uses”, cases where pesticides are applied to less common crops or for specific pest problems not widely covered by existing authorizations [15].

Moreover, cumulative risk assessments show that a small number of active substances disproportionately influence overall risk estimates under conservative assumptions [16,17]. These findings highlight the urgency of refining methodological approaches, improving prioritization criteria, and enhancing transparency in decision-making processes. The rapid evolution of scientific and technological tools further complicates the regulatory landscape. *New Approach Methodologies* (NAMs), such as in silico modeling, machine learning, and high-throughput omics technologies, are increasingly being proposed to complement or replace conventional toxicological testing. While promising, these tools raise significant challenges regarding validation, standardization, and legal acceptance [18,19,20].

This review provides a perspective on emerging challenges in pesticide risk assessment, focusing on European regulation while also referencing global disparities, emerging methodological directions, and implementation barriers. In this context, integrated toxicological assessment is defined as a multidimensional approach combining classical toxicology, biomonitoring data, high-throughput in vitro and in silico testing, and environmental exposure assessments to evaluate cumulative and low-dose effects of pesticides on human and ecological health. By integrating insights from pesticide regulation and complementary chemical safety frameworks such as REACH and CLP, this approach provides a holistic, evidence-based evaluation of risk, extending beyond traditional single-substance toxicity assessments, and can inform regulatory decision-making, policy development, and prioritization of substances for further study or restriction. By analyzing the most recent opinions of the *European Food Safety Authority* (EFSA), policy documents, and scientific literature, the review aims to identify priority areas for reform and outline evidence-based strategies for a more adaptive, integrated, and precautionary risk governance model.

In an era marked by increasing ecological challenges, climate change, and rapid scientific innovation, strengthening the scientific foundations and institutional coherence of the European regulatory system is a crucial step toward reconciling agricultural productivity, public health, and ecological sustainability.

## 2. Evidence and Regulation: State of the Art

As highlighted above (Figure 1), in the European Union, the placing on the market of PPPs is regulated under Regulation (EC) No. 1107/2009 [5], amended for microorganisms by Regulation (EU) 2022/1438 [21], and is based on a careful pre-marketing risk assessment process [22]. A substance active can only be approved when, following an exhaustive examination, no unacceptable risks are established for human health, animal health, or the environment. The assessment is founded on large dossiers submitted by producers, which have to include experimental studies conducted according to internationally accepted and standardized guidelines, as stated by Regulations (EU) No. 283/2013 [23] and No. 284/2013 [24] (amended by Regulations (EU) 2022/1439 [25] and 2022/1440 [26] for microorganisms). The decision-making criteria used by the authorities are set in the *Uniform Principles* (Regulations (EU) No. 546/2011 [27], and 2022/1441 [28] for microorganisms).

*International Uniform Chemical Information Database* (IUCLID), developed by the *European Chemicals Agency* (ECHA) in association with the *Organisation for Economic Co-operation and Development* (OECD), facilitates handling of data and allows firms to organize and submit dossiers in a harmonized format, which can be easily assessed by the *Zonal Rapporteur Member State* (zRMS) and other Member States. IUCLID ensures consistency, traceability, and comparability of ecotoxicological and toxicological data, allowing integrated and multidimensional risk assessment strategies [29,30].

Toxicological assessment involves hazard characterization and identification, *No Observed Adverse Effect Level* (NOAEL), *Lowest Observed Adverse Effect Level* (LOAEL) and *Benchmark-dose* (BMD) estimation, and application of safety factors to compute reference values such as the *Acceptable Daily Intake* (ADI) and the *Acute Reference Dose* (ARfD). Concurrently, exposure assessment considers residues in food products and allows for the setting of *Maximum Residue Limits* (MRLs), as regulated by Regulation (EC) No. 396/2005, precautionary values intended to ensure safety without necessarily indicating toxicological risk [31,32]. Ecotoxicological analysis is interested in the effect of active ingredients and formulated products on non-target organisms, terrestrial or aquatic. Acute and chronic effects are studied through laboratory, semi-field, or field tests depending on product toxicity. The no-effect concentration for each species is compared to exposure levels, taking into account the *Uniform Principles* safety factors, for obtaining ecological risk characterization. Environmental destiny of chemicals is assessed considering persistence, mobility, degradation, volatility, photolysis, hydrolysis, and metabolites, and including harmonized European modeling in order to predict concentrations in soil, surface and groundwater, sediments, and air [22,27,28,29].

**Figure 2:**
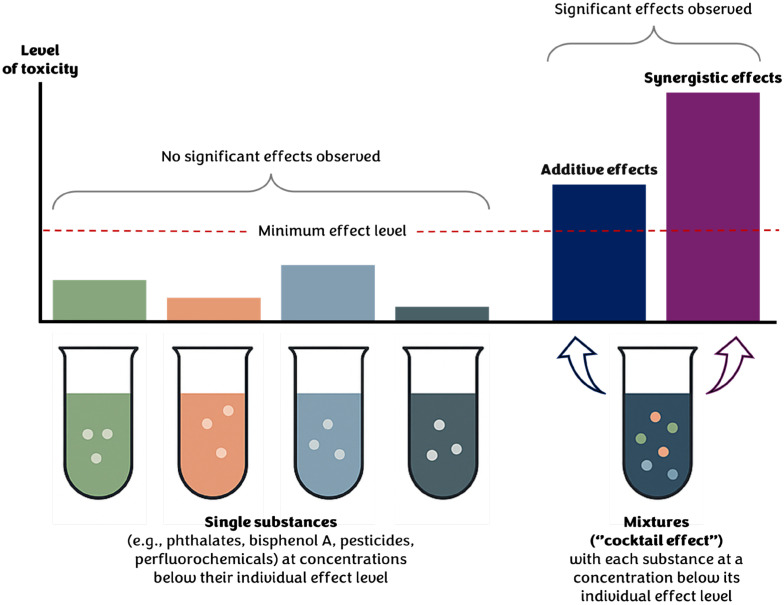
Single substances, when tested individually, produce no measurable toxic effect at concentrations below their respective effect levels. In contrast, mixtures of the same substances, each present at sub-threshold concentrations, can interact additively (blue bar) or synergistically (purple bar) (“cocktail effect”), leading to toxic effects that exceed the minimum effect level.

Despite this rigorous regulatory framework, recent research has shown severe problems with cumulative exposure and combinations of pesticides. As shown by the 2021 EFSA monitoring report, residues were detected in 96.1% of the food samples analyzed, and 28.9% of them contained more than one active substance [17]. While these figures are formally compliant with regulatory limits, they highlight the persistent challenge of multiple exposure scenarios, both in terms of aggregate exposure (i.e., exposure to the same chemical through different routes) and cumulative exposure (i.e., exposure to multiple chemicals with similar effects or modes of action), aspects that remain insufficiently considered in current risk assessment models. In particular, cumulative exposure is becoming increasingly relevant not only through diet but also via environmental and occupational pathways, as confirmed by the widespread detection of pesticide residues in surface water [33], soils [34], and human biological fluids [35]. New evidence suggests that simultaneous exposure to low doses of different pesticides may result in additive or synergistic effects [36,37]. While additive effects are much easier to address with existing data and the current risk assessment paradigm, based on chemical assessments for single chemicals, the potential for synergy, greater than additive effects, requires a deeper understanding, particularly from a mechanistic perspective, of how chemicals interact (e.g., whether the interaction occurs at the same receptor or at different receptors). Furthermore, while most studies still focus on single compounds, simultaneous exposure to multiple pesticides or to pesticides combined with other xenobiotic substances (“cocktail effects”; Figure 2) represents a much more realistic scenario [38].

Recent experimental studies further support these concerns. Developmental exposure of rats to mixtures of pesticides, such as cypermethrin (CYP) and endosulfan (END), produces effects that are significantly different from exposure to the individual pesticides. Synergistic, preventive, or antagonistic interactions were observed depending on the neurochemical or behavioral parameters analyzed, indicating that deviations from additivity are frequent [39]. In particular, the mixture induced synergistic effects on spatial memory in males and associative learning in females, while some endpoints, such as motor coordination, showed antagonistic interactions, likely due to the prevention of γ-Aminobutyric acid (GABA) increase in the cerebellum by the mixture. Moreover, neurochemical and behavioral effects were strongly sex-dependent, with differential alterations of inflammatory markers and N-methyl-D-aspartate (NMDA) receptor subunits in males and females. These findings emphasize the importance of considering combined effects of pesticides rather than single-compound effects in health risk assessment [39,40].

Additionally, the recent EFSA cumulative risk assessment published in 2024 confirmed the relevance of considering combined effects of pesticide residues targeting the thyroid, defining two *Cumulative Assessment Groups* (CAGs) for hypothyroidism and C-cell hypertrophy, hyperplasia, and neoplasia. The report highlights that a limited number of active substances disproportionately drive cumulative risk and that mixture effects should be considered in regulatory decision-making [41,42].

Despite initiatives such as the CAGs developed by EFSA, the practical incorporation of mixture toxicity into routine regulatory evaluations remains limited, often constrained by data gaps, methodological complexity, and the lack of harmonized guidance across Member States.

A critical area of ongoing debate is the identification and regulation of endocrine disruptors (EDs). Although formal identification criteria were adopted in 2018, their application in risk management decisions has been highly inconsistent [43]. Substances evaluated as EDs are not automatically subjected to substitution or phase-out unless supported by additional data, and classification under the CLP Regulation often lags behind hazard identification. While Commission Delegated Regulation (EU) 2023/707, introducing the *Persistent*, *Mobile and Toxic*/*very Persistent and very Mobile* (PMT/vPvM) categories, represent an evolution toward a more systemic hazard-based approach, its practical implementation requires the development of operational guidance, clear threshold criteria for classification, prioritization tools, and cross-authority coordination to integrate PMT/vPvM measures into risk management decisions [44].

Another important aspect concerns the use of emergency authorizations under Article 53 of Regulation (EC) No. 1107/2009 [5]. Initially intended to address specific and time-limited threats to crop protection, this regulatory tool remains a key instrument for managing unforeseen phytosanitary challenges. Between 2017 and 2021, Member States granted over 600 such authorizations annually, with a significant proportion renewed across multiple years [13,14]. While these authorizations respond to practical agricultural needs, their frequency highlights the complex interplay between pest control demands, availability of effective alternatives, and the pace of regulatory and agronomic innovation. Strengthening the interface between risk assessment, comparative evaluation, and long-term transition strategies may help ensure that emergency measures continue to fulfill their original purpose while supporting more sustainable plant protection approaches.

Despite the scientific and political recognition of these issues, the regulatory paradigm remains dominated by a model based on the assessment of individual substances, which struggles to reflect the complexity of the real world, including the toxicity of mixtures, low-dose effects, and the dynamic interactions between environmental and human health factors. Indeed, current European regulations, such as the *Mixture Assessment Factor* (MAF) approach, do not fully address the risks arising from cumulative exposures and mixtures of substances [45,46]. Furthermore, the guidelines for their assessment are not yet harmonized across the various regulatory sectors, resulting in a significant gap in risk management. Currently used modeling tools also fail to adequately represent the ecological complexity of the real world, limiting the ability to reliably estimate overall environmental impacts. Despite increasing calls for a shift towards integrated assessment frameworks, including cumulative risk models, high-throughput screening tools and population-level biomonitoring programs, such approaches are not yet structurally embedded in EU practice. Comparative insights from other regulatory systems, such as the *Environmental Protection Agency* (EPA—United States) or the *Pest Management Regulatory Agency* (PMRA—Canada), may offer useful contrasts in how cumulative risk and endocrine disruption are operationally addressed [47,48,49,50]. In particular, the EPA provides a structured approach to pesticide risk assessment that can serve as a useful comparison. The agency evaluates both ecological and human health risks through multi-step processes, including hazard identification, dose–response assessment, exposure analysis, and risk characterization. Cumulative risk assessment is explicitly incorporated, considering simultaneous exposures to multiple pesticides with common mechanisms of toxicity across dietary, environmental, and residential pathways. Additionally, the EPA employs standardized models and technical guidelines to predict environmental concentrations and potential effects on non-target organisms [51].

Closing these gaps will require not only scientific advancement, but also institutional reform, more open implementation paths, and more investment in data and knowledge infrastructures with the potential to support integrated, multidimensional risk analysis. To take decisive steps in this direction, a more visionary, concerted, and ambitious approach is needed. First, monitoring protocols must be coordinated and strengthened at the regional, national, and EU levels, including not only all active substances but also the most significant metabolites and transformation products. To this end, it is necessary to develop harmonized risk assessment methodologies capable of addressing the combined and additive/synergistic effects of pesticide mixtures, going beyond the traditional approach, applied in the REACH Regulation, which focuses almost exclusively on individual compounds. To this end, it is necessary to implement existing regulatory provisions with workplace exposure scenarios. In parallel, research efforts must be intensified to investigate the cumulative, chronic, and sub-lethal impacts of pesticides, particularly on non-target organisms, pollinators, and soil microbiota, which play a key role in ecosystem stability but are often overlooked in conventional assessments. Integrating advanced analytical techniques, such as biomonitoring, omics approaches, and remote sensing, could substantially enhance the accuracy and temporal resolution of environmental data. At the same time, it is necessary to ensure environmental surveys, to obtain an accurate and comprehensive photography of the contamination situation. This is accompanied by the need to strengthen resistance monitoring programs to quickly identify any emerging outbreaks and specify more resilient management and rotation practices. Finally, a tangible measure to promote more responsible use would be the enforcement of mandatory phytosanitary prescriptions prepared by certified experts, could be complemented by farmer training, public awareness campaigns, and incentive schemes for integrated pest management practices. This measure would enable the regulation and rationalization of the employment of plant protection products, as well as ensuring increased safety for the environment and public health.

## 3. Emerging Critical Issues in Risk Assessment

Current regulatory frameworks for pesticide risk assessment remain grounded in a reductionist approach, relying heavily on standardized testing of individual active substances. While this has historically facilitated harmonization and comparability, it falls short in addressing the systemic, dynamic, and context-specific nature of real-world exposure [52,53].

Several European agencies are responsible for assessing environmental and health risks associated with both occupational and non-occupational exposure to pesticides. The EFSA provides scientific evaluations of risks to human health, animals, and the environment, including residue levels in food and feed. The European Commission, based on EFSA assessments, authorizes active substances and establishes maximum residue limits, while Member States implement these regulations at the national level and monitor pesticide use. The ECHA supports safe chemical management, including data relevant to pesticide risk assessment. Despite these roles, gaps remain, such as limited integration of cumulative exposure scenarios, inconsistent risk management of endocrine disruptors, and fragmented coordination across Member States. Italy represents an excellent model of virtuous management and coordination of PPPs. In fact, in Italy, several authorities are involved in pesticide regulation. The *Ministry of Health* is the competent authority for the authorization of PPPs and for the monitoring and enforcement of regulations relating to human health, while the *Ministry of Agriculture*, *Food Sovereignty and Forests*, particularly through the *National Phytosanitary Service*, supervises the use of pesticides in crop protection. The *Ministry of the Environment*, Italian *Institute for Environmental Protection and Research* (ISPRA) and regional health authorities are responsible for environmental monitoring and risk assessment. All these bodies cooperate with the European Commission, EFSA and other European organizations, such as the *European and Mediterranean Plant Protection Organization* (EPPO) and *European Minor Uses Coordination Facility* (MUCF), to ensure compliance with EU standards.

This section discusses three interconnected challenges, cumulative exposure, low-dose effects, and environmental persistence, that illustrate the limits of current paradigms and the need for integrated adaptive methodologies.

### 3.1. Cumulative Exposure and Chemical Mixtures

In both occupational and environmental settings, exposure to pesticides occurs through complex chemical mixtures, yet current risk assessment procedures still prioritize single-compound evaluations [54]. These regulatory and operational challenges intersect with methodological complexities in risk assessment. The development of CAGs by EFSA represents an important conceptual advance, particularly for organophosphates and triazole fungicides, but these methods remain largely non-binding and inconsistently applied in regulatory decision-making. The derivation of CAGs relies on grouping substances based on shared mechanisms of toxicity, chemical structure, or target organs, with thresholds set to reflect combined risk potential. However, the operational application of these thresholds is limited by the availability of co-exposure data, the need for validated models for interaction effects, and uncertainties in extrapolating to different populations or exposure scenarios [3,16,55].

Integrating cumulative exposure into EU regulatory practice would require not only adoption of group-based thresholds, but also explicit guidance on threshold calculation, monitoring of high-risk substance clusters, and implementation of post-market surveillance to verify compliance and effectiveness. Agencies like the U.S. EPA have developed cumulative risk frameworks based on shared mechanisms of toxicity, albeit with operational limitations. The EU could benefit from a more explicit adoption of group-based regulatory thresholds, combined with targeted monitoring of high-risk substance clusters. A shift towards this model would also require greater data sharing, interoperable exposure databases, and post-market surveillance mechanisms. Overall, clearer methodological guidance and enhanced data infrastructure are essential to translate CAG-based thresholds into actionable regulatory decisions.

### 3.2. Low-Dose Effects and Non-Monotonic Dose–Response Curves

A key principle of chemical risk assessment is the protection of populations from adverse effects, rather than the suppression of all measurable biological changes, which are part of normal organismal variability. Therefore, the observation of biological responses below established reference points, such as NOAEL, LOAEL and BMD, is not in itself indicative of regulatory failure. However, such sub-threshold responses acquire toxicological relevance in contexts of aggregate or cumulative exposure, where multiple simultaneous exposures, to the same substance via different routes or to different substances sharing similar modes of action, may combine such that the net response exceeds the threshold into adversity. Evidence of low-dose biological activity for certain classes of pesticides (e.g., some neonicotinoids, organophosphates, and pyrethroids) highlights the need to integrate mechanistic information, like *Mode of Action* (MOA) and *Adverse Outcome Pathways* (AOPs), weight-of-evidence evaluation, and exposure context into assessment frameworks, rather than discarding the classical toxicological paradigm outright. The 2021 EFSA Scientific Committee Opinion [56,57] on *non-monotonic dose responses* (NMDRs) provides an operational analysis of when apparent NMDRs warrant further consideration in human health risk assessment. The Opinion offers practical criteria, including evaluation of biological plausibility, proximity to adversity, and availability of mechanistic MOA evidence, to guide assessors. When these criteria indicate potential regulatory relevance, targeted additional low-dose analyses or a refined uncertainty evaluations are recommended.

Furthermore, some pesticides exhibit a NMDR where lower doses give rise to a stronger or qualitatively different response than higher doses [58]. For example, for endocrine disruptors, non-monotonicity can be observed through mechanisms such as receptor down-regulation, selective activation of signaling pathways in response to varying doses, or feedback loops in hormonal systems. These non-monotonic dose–response relationships complicate the definition of health reference values because they undermine the original toxicological notion of monotonicity and hence well-defined effect thresholds. NMDRs, therefore, represent a significant challenge in risk assessment as they inhibit the reliable determination of NOAELs and margins of safety and illustrate the limitations of the current *Organization for Economic Co-operation and Development* (OECD) Test Guidelines in capturing all these complexities in their assessments and thus limit their ability to inform hazard identification. In other words, this implies that current OECD guidelines, which are primarily designed to identify monotonic dose–response relationships, may miss adverse effects that occur only at low or intermediate doses, resulting in a likely underestimation of the hazard potential.

To address these gaps, EU regulatory science would benefit from incorporating low-dose and NMDR data into risk assessments, revising testing protocols, and embracing mechanistic and omics-based evidence. Specifically, as will be discussed in greater detail in the following paragraphs, omics technologies, including genomics, transcriptomics, proteomics, metabolomics, and epigenomics, can provide mechanistic insights into molecular pathways affected by pesticide exposure, identify early biomarkers of effect, and reveal systemic or transgenerational impacts that classical approaches cannot detect. Integration of these datasets into risk assessment frameworks allows for the identification AOPs, supports probabilistic modeling, and strengthens predictive capabilities for human and ecological health. Moreover, multi-omics approaches can be combined with computational tools, such as *quantitative structure–activity relationship* (QSAR) and *physiologically based pharmacokinetic* (PBPK) models, to improve mechanistic understanding and guide the design of safer chemicals.

Shifting from fixed-threshold models toward probabilistic and systems-based risk evaluation approaches could substantially improve the realism and reliability of pesticide regulation [59].

### 3.3. Environmental Persistence and Bioaccumulation Under Climate Change Scenarios

**Figure 3:**
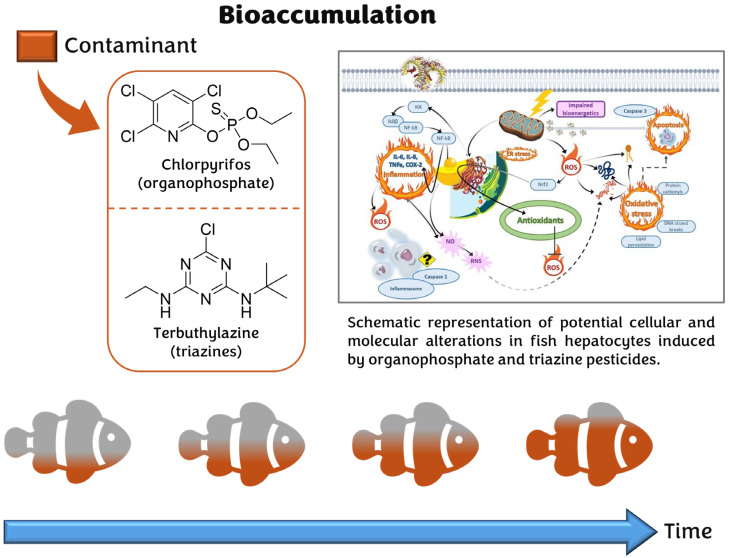
Progressive bioaccumulation of contaminant over time due to their persistence and lipophilicity. Fish, as key inhabitants of aquatic ecosystems, are a major focus in the assessment of the chronic toxicity of pesticides, particularly because of their potential for bioaccumulation and bioconcentration. These processes may occur through dermal, respiratory, or oral routes, with pesticide residues entering the organism either by direct diffusion from water or through ingestion of contaminated prey, leading to biomagnification. Among the pesticides most frequently detected in aquatic environments worldwide, including Central and Eastern Europe, are organophosphates (e.g., chlorpyrifos, glyphosate), phenylureas (e.g., chlorotoluron), triazines (e.g., terbuthylazine), and neonicotinoids. Fish can accumulate these compounds more efficiently than mollusks, and once accumulated, they may impair redox balance and cause toxic effects such as oxidative stress, cellular damage, inflammation, and altered immune and neuroendocrine functions [67,68]. The diagram of potential cellular and molecular alterations shown in the figure is adapted from [67].

Many pesticides are highly persistent in the environment and prone to bioaccumulation, impacting soils, aquatic ecosystems, and entire food webs. Their persistence and lipophilicity facilitate accumulation through dermal, respiratory, and dietary exposure, often leading to internal concentrations exceeding those in the surrounding environment, although this does not necessarily indicate a health risk, further assessment is needed considering additional factors such as toxicity and duration of exposure [60] (Figure 3).

Despite these risks, current monitoring and assessment programs remain fragmented and unevenly applied across EU Member States. Ecotoxicological indicators, such as shifts in macroinvertebrate populations or changes in soil microbiomes, are still underutilized, even though they can serve as early warning signals of ecological disruption [61,62].

In addition, a growing body of research demonstrates that climate change interacts with chemical pollution, affecting pesticide persistence, bioavailability, and toxicity. Changes in temperature, precipitation, and extreme weather events can modify chemical occurrence, alter uptake and elimination in organisms, and influence trophic transfer within ecosystems. For example, higher temperatures can increase chemical volatility and degradation rates, while also modulating bioaccumulation and toxicity through oxidative stress, metabolic interference, and disruption of species adaptation mechanisms [63,64,65]. Extreme weather events may further exacerbate chemical risks by breaching biological adaptation thresholds, while temperature fluctuations can produce nonlinear or sex-dependent effects on organisms. These factors introduce significant uncertainty into ecological risk assessment, highlighting the need for frameworks that quantitatively integrate climate-driven changes in chemical occurrence, bioaccumulation, and toxicity [66].

Climate change profoundly influences pest and pathogen dynamics, reshaping their geographic distribution, life cycles, population growth, and interactions with crops. Rising temperatures, altered precipitation patterns, and higher atmospheric CO_2_ levels can intensify pest pressure and extend growing seasons. At the same time, these factors may reduce pesticide efficacy, often requiring more frequent or larger applications. Sustainable crop management strategies, such as crop rotations, remain essential to mitigate pest prevalence and decrease reliance on chemical controls. Tools like CropRota integrate agronomic criteria with historical crop data to optimize rotations at field, farm, or regional scales, supporting long-term planning [69]. In parallel, process-based crop–pest models such as Pest-EPIC allow the simulation of crop–pest interactions in both conventional and organic systems, helping assess yield gaps and pesticide requirements under current and projected climatic conditions [70]. The use of such models not only strengthens the robustness of risk assessments but also contributes to the design of more effective adaptation strategies under evolving agro-ecological scenarios.

Addressing the uncertainties inherent in climate–crop–pest interactions requires holistic, scenario-based approaches that combine predictive models with empirical data on pest populations, pesticide use, and crop rotation practices. This integration enables researchers, farmers, and policymakers to develop adaptive strategies that maximize crop production, minimize environmental impacts, and enhance system resilience. Nonetheless, important knowledge gaps persist, particularly regarding pesticide efficacy under changing climate conditions, shifts in pest populations, and the interactions between climate change and pesticide persistence and degradation. Understanding how elevated temperatures, increased CO_2_ levels, and altered precipitation influence pest life cycles, chemical resistance, and the effectiveness of control measures, including biological control agents, is critical. It is equally important to evaluate whether current regulatory frameworks are flexible enough to accommodate climate-related uncertainties and support adaptive agricultural practices. Through targeted research and strong collaboration among scientists, farmers, and policymakers, sustainable, climate-resilient, and environmentally conscious pest management strategies can be achieved, ensuring safe and effective food production in the face of global change [69,70].

The introduction of hazard categories for PMT (*Persistent*, *Mobile and Toxic*) substances under Commission Delegated Regulation (EU) 2023/707 offers a new opportunity to address these challenges [44]. However, fully operationalizing these classifications in EU risk assessment requires improved methodological frameworks, integration of environmental fate data, adoption of passive sampling technologies, and bioindicator-based monitoring. Implementing these measures would enhance the ecological relevance of pesticide risk evaluation and support more sustainable regulatory decisions.

### 3.4. Endocrine Disruptors and Reprotoxicants

In recent years, growing attention has been directed toward *endocrine disruptors* (EDs) and *reprotoxic* (Repr.) substances found in plant protection products [71,72,73]. Considering these concerns, it is essential to map which substances used as active ingredients in plant protection products have been associated with endocrine-disrupting effects [74,75]. Table 1 identifies the pesticides with endocrine-disrupting effects according to the *Endocrine Disruptor List* [76] and most recent EFSA reports [77,78]. These evidence-based lists of (potential) EDs have been compiled from existing sources and published by the national authorities of Belgium (*Federal Public Service—Health*, *Food Chain Safety and Environment* and *National Environment and Health Action Plan*), Denmark (*Danish Environmental Protection Agency*), France (*French Agency for Food*, *Environmental and Occupational Health & Safety—ANSES* and *French Ministry for the Ecological and Inclusive Transition*), the Netherlands (*The Ministry of Infrastructure and Water Management*), Spain (*General Directorate for Environmental Quality and Assessment—Ministry for the Ecological Transition and Demographic Challenge* and *General Directorate for Public Health—Ministry of Health*), and Sweden (*Swedish Chemicals Agency*—KEMI). The lists are updated at least twice a year. These compounds represent significant hazards to human health and ecosystems, as they can disrupt endocrine function (EDs) or cause reproductive toxicity (Repr.), even at very low concentrations, especially during sensitive developmental windows. Such effects are frequently associated with *non-monotonic dose–response* (NMDR) relationships and transgenerational outcomes, which are poorly captured by conventional toxicological models based on linear assumptions and fixed thresholds like NOAEL and LOAEL. Experimental data from both in vivo and in vitro studies show that substances such as chlorpyrifos, linuron, and vinclozolin can induce endocrine or developmental toxicity at exposure levels within current regulatory safety margins, indicating a consistent underestimation of risk in existing frameworks [79,80,81].

Current scientific understanding indicates that EDs may act as agonists or antagonists of key hormone receptors, such as steroid or thyroid receptors, and can interfere at multiple levels, including hormone synthesis, secretion, transport, binding, action, and elimination. These disruptions may not manifest as acute toxicity but can result in long-term or delayed adverse effects, including reproductive disorders, certain types of cancer, metabolic conditions such as obesity and diabetes, and cardiovascular disease. Moreover, environmental exposure, even at low doses, can produce population-level impacts with potential ecological repercussions, especially in wildlife, due to the capacity of these compounds for bioaccumulation [82,83].

#### 3.4.1. Evolving Regulatory Criteria and Implementation Gaps

Regulation (EC) No. 1107/2009 provides for the automatic exclusion from the market of active substances classified as *endocrine disruptors* or *reprotoxic* (Category 1A or 1B under the CLP Regulation), unless exposure is deemed negligible [5]. Official scientific criteria for ED identification were established with Regulation (EU) 2018/605 (plant protection products) [73] and Regulation (EU) 2017/2100 (biocidal products) [84], aligned with the *World Health Organization* (WHO) definition [85]. These criteria require three elements: (1) evidence of an adverse effect, (2) identification of an endocrine mode of action, and (3) a demonstrated causal link between the two. Despite this regulatory progress, implementation remains uneven. A significant number of substances with documented or suspected endocrine-disrupting activity are still not formally classified under the CLP Regulation, resulting in delays in substitution procedures and fragmented regulatory responses across Member States.

Some EDs are regulated because of their general toxicity, carcinogenicity, or reproductive toxicity, even though their endocrine-disrupting effects occur at doses higher than those that cause these other effects and are therefore not the primary driver of regulation. However, is still important to understand the risks posed by EDs under low-dose, long-term, and combined exposure conditions, as current toxicological models and testing strategies are insufficient to capture the full complexity of endocrine disruption, highlighting the need for precautionary and mechanistic approaches.

The recent adoption of Commission Delegated Regulation (EU) 2023/707 [44], which amends the CLP Regulation by introducing new hazard classes [86], including EDs (for both human health and the environment), *Persistent*, *Bioaccumulative and Toxic/very Persistent and very Bioaccumulative* (PBT/vPvB), and *Persistent*, *Mobile and Toxic/very Persistent and very Mobile* (PMT/vPvM), represents a significant step toward a more integrated and precautionary risk assessment framework. These classifications have become legally binding as of 1 May 2025 for newly registered substances and will become legally binding from 1 November 2026 for substances already on the market. Their implementation is expected to influence authorization re-evaluations, safety data sheet labelling, national risk management systems, and substitution procedures under the “candidates for substitution” provisions of Regulation (EC) No. 1107/2009 [5].

Evidence from EFSA conclusions, the *European Chemicals Agency* (ECHA), and peer-reviewed studies confirm that many substances previously approved in the EU exhibit endocrine or reproductive toxicity, and several remain in use outside the EU, highlighting the need for harmonized international monitoring and trade alignment.

Table 2 presents a selection of active substances with well-documented endocrine-disrupting or reprotoxic effects, Prochloraz, Chlorpyrifos, Epoxiconazole, Vinclozolin, Linuron, Mancozeb, and Carbendazim, together with their chemical class, regulatory status, mechanisms of action, and CLP classification.

Notably, several of these substances, such as Epoxiconazole, Linuron, and Carbendazim, are officially classified under the CLP Regulation as *Repr*. Category 1B and are listed as candidates for substitution, indicating a regulatory trajectory towards the adoption of safer alternatives. Furthermore, epoxiconazole is considered PBT by most data submitters in the ECHA database, although this classification has not yet been formalized under the CLP [87,88,89]. Chlorpyrifos, by contrast, is currently under assessment for potential PBT properties, in addition to its documented neurotoxic and suspected endocrine-disrupting effects. In 2019, EFSA identified concerns for genotoxicity and developmental neurotoxicity, supported by epidemiological evidence, and concluded that the substance does not meet the approval criteria in the EU. Following Council Decision (EU) 2021/592 of 7 April 2021 [90] on the submission, on behalf of the European Union, of a proposal for the listing of chlorpyrifos in Annex A to the Stockholm Convention on Persistent Organic Pollutants (POPs), Chlorpyrifos is officially recognised POP (Substances proposed as POPs) and has recently been added to Annex A (Elimination) of the Convention [91]. For these reasons Chlorpyrifos is no longer approved in the EU since 2020 (Commission Implementing Regulation (EU) 2020/18 of 10 January 2020 [92]) and is not currently listed as a candidate for substitution [93].

**Table 2:** Overview of active substances in plant protection products, mechanisms of actions in human and animals, observed effects and their regulatory status. ^a^ Based on scientific literature; ^b^ Verified on ECHA C&L Inventory—https://echa.europa.eu/information-on-chemicals/cl-inventory-database (accessed on 21 April 2025); ^c^ Verified on The EU Pesticides Database—https://food.ec.europa.eu/plants/pesticides/eu-pesticides-database_en (accessed on 21 April 2025). ED: endocrine disruptors; Repr: reprotoxic; ✔: Harmonized classification—Annex VI of Regulation (EC) No. 1272/2008 (CLP Regulation); ✖: not classified.

Active Substance	Chemical Class (Type)	Mechanism of Action in Human and Animals	Observed Effects	Category ^a^	Regulatory Classification (EU) ^b^	EU Status ^c^
Carbendazim	Benzimidazole (fungicide)	Inhibition of meiosis	Malformations and spermatogenesis toxicity [94]	Repr. 1B	✔ Repr. 1B (CLP) + ✖ ED (not classified)	Not approved (Expiry: 30 November 2014)
Chlorpyrifos	Organophosphorus (insecticide)	Acetylcholinesterase inhibition, neuroendocrine alterations	Delayed brain development [95]	Suspected ED, Neurotoxic	✖ ED (not classified)	Not approved (Expiry: 16 January 2020)
Epoxiconazole	Triazole (fungicide)	Aromatase inhibition	Fetal and developmental toxicity [96]	Repr. 1B, Repr. 2, suspected ED	✔ Repr. 1B and 2 (CLP) + ✖ ED (not classified)	Not approved (Expiry: 30 April 2020)
Linuron	Phenylurea (herbicide)	Androgenic antagonism	Sexual development disorders [97]	Repr. 1B, Repr. 2, suspected ED	✔ Repr. 1B and 2 (CLP) + ✖ ED (not classified)	Not approved (Expiry: 3 March 2017)
Mancozeb	Dithiocarbamate (fungicide)	Thyroid interference	Neurotoxicity, thyroid dysfunctions [98]	Repr. 1B, suspected ED	✔ Repr. 1B (CLP) + ✖ ED (not classified)	Not approved (Expiry: 4 January 2021)
Prochloraz	Imidazole (fungicide)	Inhibition of steroidogenic enzymes	Testicular dysfunction, thyroid alterations [99]	Suspected Repr. 1B and ED	✖ Repr. 1B and ED (not classified)	Not approved (Expiry: 31 December 2021)
Vinclozolin	Dicarboximide (fungicide)	Androgen receptor antagonism	Transgenerational effects [100]	Repr. 1B	✔ Repr. 1B (CLP) + ✖ ED (not classified)	Banned

For Prochloraz, EFSA published a Reasoned Opinion in 2023 on the review of *maximum residue levels* (MRLs) under Article 12 of Regulation (EC) No. 396/2005, highlighting consumer risks that led the European Commission to lower the MRLs to the *limit of determination* (LOD) [32]. Since 31 December 2021, Prochloraz has no longer been approved in the EU and is also listed as a candidate for substitution [101].

Regarding Mancozeb, EFSA concluded in 2019 that the substance did not meet the approval criteria under Article 4 of Regulation (EC) No. 1107/2009, based on its potential as an endocrine disruptor for humans and non-target organisms, risks from non-dietary exposure, and adverse effects on terrestrial fauna. As a result, its EU approval was not renewed; however, it is not currently listed as a candidate for substitution [102].

For Vinclozolin, although no recent EFSA opinions with the same level of detail have been identified, longstanding evidence of its endocrine-disrupting properties and reproductive toxicity has already led to revocations or bans within the EU [100].

These examples clearly illustrate the gap between robust scientific evidence and formal regulatory action, where classification of endocrine disruptors often requires particularly stringent criteria. Such gaps highlight the urgent need for stronger integration of EFSA peer reviews, the joint ECHA/EFSA guidance on endocrine disruptors, and OECD testing standards into regulatory decision-making. Without this, regulatory frameworks risk lagging behind scientific advances, undermining the protection of human and environmental health.

An interesting case currently under evaluation is acetamiprid. It is a neonicotinoid agonist of nicotinic acetylcholine receptors (nAChRs) with systemic and translaminar action. Currently, through Commission Implementing Regulation (EU) 2018/113 [103], acetamiprid is approved until 28 February 2033. However, in November 2021, EFSA recommended evaluating the endocrine-disrupting properties of acetamiprid in line with the criteria established by Regulation (EU) 2018/605 [73,104]. Furthermore, in its report of 15 May 2024, EFSA highlighted gaps in available studies regarding developmental neurotoxicity [105]. Consequently, it was not possible to establish with certainty whether acetamiprid still meets the approval criteria set out in Article 4 of Regulation (EC) No. 1107/2009 [5] with regard to its developmental neurotoxicity, as well as for its endocrine-disrupting properties, for which no assessment is available. Therefore, a review of the authorization under Article 21 of Regulation (EC) No. 1107/2009 is ongoing from December 2024 to assess developmental neurotoxicity and its endocrine-disrupting properties [106]. The EFSA report of 30 September 2025 expresses a favorable opinion on the new tests on acetamiprid, with some uncertainties considering the completeness of the data set. It recommends additional tests to investigate endocrine activity in non-mammalian species and a review of the scientific literature to assess the effects on humans and non-target organisms [107].

Overall, these cases underscore the need for a dynamic, precautionary, and evidence-responsive regulatory approach, capable of rapidly incorporating new scientific insights across the EU.

#### 3.4.2. Implications for Risk Assessment: Toward a Science-Based Paradigm Shift

The specific characteristics of EDs and Repr. substances have significant implications for the future of pesticide risk assessment, requiring a shift from traditional, linear, single-compound models to a more integrated approach. A growing body of literature shows that many EDs exhibit adverse effects at extremely low concentrations, often below conventional safety thresholds such as NOAELs [9], challenging the regulatory reliance on threshold-based toxicology and, consequently, the associated risk assessment. Non-monotonic dose–response curves, where lower doses may cause stronger or qualitatively different effects than higher ones, complicate the establishment of reliable reference values and safety factors.

Emerging data on epigenetic and transgenerational effects, as observed in substances like vinclozolin and prochloraz [92,93], indicate that even transient exposures during sensitive life stages can produce heritable molecular and physiological changes. In particular, these transgenerational outcomes have been documented across multiple generations in both animal models and epidemiological studies, demonstrating effects on reproductive health, metabolic regulation, and neurodevelopment [108,109,110]. Such findings underscore the importance of incorporating multi-generational endpoints into risk assessment frameworks, as standard one-generation tests may underestimate long-term hazards [111,112]. These findings suggest a need to expand both the temporal and biological scope of toxicological evaluations.

Standard regulatory testing frameworks also show critical methodological limitations: many validated protocols do not include endpoints sensitive to endocrine disruption, such as steroidogenesis, early thyroid signaling, or hypothalamic–pituitary–gonadal axis function, resulting in frequently missed receptor-mediated activity (e.g., estrogenic or anti-androgenic effects). For example, recent studies demonstrate that glyphosate may inhibit aromatase, reducing estrogen synthesis, while neonicotinoids like thiacloprid and imidacloprid can act on estrogen receptors, potentially impairing reproductive function even at low exposure levels [113,114].

Additionally, oxidative stress, exacerbated by poor diet and cumulative environmental exposures, may act as a co-factor in endocrine disruption, particularly by triggering epigenetic modifications in germ cells and developing tissues [115]. These cross-cutting mechanisms emphasize the need for an integrated, systems-level understanding that integrates biochemical, physiological, environmental, and social determinants of risk.

## 4. Regulatory Implementation Proposals

Based on the gaps and challenges identified in previous sections, the following operational proposals aim to strengthen the EU regulatory framework for endocrine disruptors and reproductive toxicants. These measures focus on improving scientific assessment, harmonizing regulatory practices, and enhancing transparency and precaution in pesticide governance.

- Retrospective re-evaluation of legacy substances: Substances approved before 2018, prior to the adoption of harmonized ED criteria, should undergo re-assessment under the updated scientific framework. This is essential to ensure consistency across the EU market and prevent the persistence of hazardous compounds based on outdated evaluations.
- Mandatory inclusion of ED-specific test guidelines: Regulatory submissions should require the systematic application of OECD testing guidelines specifically designed to detect endocrine and reproductive effects, such as Test Guidelines (TG) 440, 443, 456, and 458 [116,117,118,119]. These should be complemented by mechanistic and receptor-binding assays, where relevant.
- Modernized, transparent data systems: Platforms like OpenFoodTox, IUCLID, and the substance database of ECHA should be enhanced with standardized visual flags (e.g., ED, Repr. 1B, PMT), searchable endpoints, and dynamic links to scientific references. These improvements would allow regulators, researchers, and stakeholders to quickly access hazard classifications, support comparative assessments and substitution, and improve transparency for consumers.
- Regulatory harmonization across frameworks: Increased alignment is needed between Regulation (EC) No. 1107/2009 (plant protection products), REACH (industrial chemicals), and the CLP Regulation, particularly in terms of classification criteria, data requirements, hazard communication and workplace exposure. This would reduce redundancy, promote consistent decision-making, and avoid regulatory “blind spots” between sectors.
- A new hazard class is proposed for chemicals that have harmful effects on the immune system (immunotoxic substances), which would be incorporated into the CLP Regulation [7] to define their classification and labeling. In fact, various studies conducted in recent times showed that exposure to fungicides, herbicides, and insecticides is associated with an enormous number of diseases, including inflammatory diseases, rheumatoid arthritis, metabolic syndrome, cancer, respiratory diseases, nervous system diseases, and alteration in various organ systems [120,121]. These disorders are strongly associated with immunological dysfunction, one of the principle aims of the toxic activity of pesticides. Immuno-toxic substances, to which many pesticides belong, are capable of inhibiting innate and acquired immune functions, causing toxicity of varied severity to the organism. Tests conducted during the past few years on cells, animals, and human subjects have proved that pesticides substantially alter humoral and cell-mediated immunity [122,123,124]. These medications can specifically target single cells of the immune system, such as T lymphocytes, B lymphocytes, natural killer (NK) cells, and macrophages, inhibiting their survival, growth, and functional capability. Effects most frequently observed are apoptosis, or programmed cell death, cell cycle inhibition, DNA damage, and alteration of intracellular signal transduction mechanisms. On the molecular level, pesticides interfere with vital biological pathways and induce oxidative stress, mitochondrial injury, and endoplasmic reticulum stress [125,126]. These pathways lead to modifications of the expression of key factors like nuclear factor kappa B (NF-κB), pro-inflammatory cytokines including inter-leukin-6 (IL-6), interleukin-8 (IL-8), and interferon-gamma (IFN-γ), and anti-inflammatory mediators like interleukin-10 (IL-10) [127,128]. It has been noted that widely used or banned pesticides, like organophosphorus compounds (OPs), carbamates, pyrethroids, and other chemicals, have been associated with reduced antibody formation, inhibition of virus-induced B-cell differentiation into plasma cells, decreased T-lymphocyte proliferation, and repressed lytic activity of NK cells [120,121,129]. Certain pesticides have caused decreased phagocytosis, oxidative injury, and reorganization of cytokine production in macrophages that are essential for anti-viral and inflammatory function. These results, both in models and in studies of exposed populations, such as farmers, confirm the high immuno-toxic potential of pesticides. It has been observed that during peak agricultural seasons, B and T lymphocytes among workers exhibit higher levels of DNA damage than unexposed populations, posing a real risk to human health [130,131]. These immune system alterations can have long-term consequences, resulting in the onset of chronic diseases and increased susceptibility to infections, cancer, and inflammatory disorders. However, pesticide exposure can affect not only adults, particularly workers, but also children. Studies demonstrate an association between residential pesticide exposure in children and adolescents and the onset of leukemia, the most common pediatric cancer. In addition to leukemia, pesticide exposure increases the risk of non-Hodgkin’s lymphoma (NHL), amyotrophic lateral sclerosis, asthmatic events, type 2 diabetes, and neurodegenerative diseases such as Alzheimer’s and Parkinson’s [128,132,133,134].
  On this basis, which adds to that about endocrine-disrupting and reprotoxic activities of some pesticides, it is a question of priority to improve new assessments based on immuno-toxicological markers, capable of identifying the risks early on and guiding the use of more restrictive regulation for pesticide use. At the same time, efforts must be made to encourage the development of less hazardous substitutes and integrated control methods in a manner that will protect public health without jeopardizing agricultural production.

Meeting the challenge of EDs, Repr., and immunotoxics requires a precautionary, integrated approach bridging toxicology, systems biology, environmental monitoring, and global regulatory cooperation, representing a paradigm shift toward more responsive, anticipatory, and transparent pesticide governance.

## 5. Future Scientific Directions: Predictive Toxicology and Innovative Approaches

Among the future outlooks, particular attention should be paid to predictive toxicology and the development of NAMs. To this end, not only financial resources, but also operational and expertise are needed to validate in vitro and in silico tools (such as omics technologies, QSARs, and machine learning-based classifiers) so that they can reliably predict EDs and reproductive toxicity more efficiently and with fewer animal studies.

Validation programs must go beyond mere technical reproducibility and be able to explicitly link molecular and cellular signals measured in vitro in order to establish biological pathways or *Adverse Outcome Pathways* (AOPs), establishing quantitative decision thresholds capable of distinguishing adaptive changes from potentially harmful ones [135,136]. This will require well-characterized reference sets, multicenter reproducibility trials, transparent performance metrics, and early dialogue with regulators and stakeholders to foster acceptance and implementation.

### 5.1. Human Biomonitoring and Real-World Exposure

*Human Biomonitoring* (HBM) represents a fundamental advancement in assessing pesticide exposure, overcoming the limitations of predictive models based solely on laboratory data and dietary intake. The analysis of biological fluids, such as blood, urine, hair, or breast milk, enables the direct detection of specific metabolites, offering concrete evidence of both the presence and concentration of contaminants in the body [137]. Importantly, these biomonitoring results can be directly linked to trade patterns and climate change impacts, as changes in pesticide use, environmental distribution, and degradation influence human exposure profiles.

Among the commonly used biomarkers for estimating population exposure are various urinary metabolites, such as 3-phenoxybenzoic acid (3PBA), a common metabolite of several pyrethroids; 4-fluoro-3-phenoxybenzoic acid (FPBA), specific to cyfluthrin; 3-(2,2-dibromovinyl)-2,2-dimethylcyclopropane carboxylic acid (DBCA), specific to deltamethrin; cis-/trans-3-(2,2-dichlorovinyl)-2,2-dimethylcyclopropane-1-carboxylic acid (DCCA); and dialkyl phosphates, including dimethyl phosphate (DMP), diethyl phosphate (DEP), O,O-dimethyl thiophosphate (DMTP), O,O-diethylthiophosphate (DETP), O,O-dimethyl dithiophosphate (DMDTP), and O,O-diethyl dithiophosphate (DEDTP), specific for organophosphate pesticides [138,139,140,141,142]. These biomarkers provide evidence of how climate-driven changes in chemical persistence, bioavailability, and international pesticide trade influence internal doses in human populations.

HBM allows for the identification of new chemical exposures, monitoring of temporal trends, and analysis of exposure distribution across various population groups, particularly vulnerable ones (e.g., children) [143,144]. It is a methodology capable of linking potential external exposure, often described or estimated in worst-case scenarios, to actual internal concentrations, thereby providing reliable information on the extent and intensity of exposure, to be combined with toxicological data for risk characterization [145,146,147].

HBM plays a decisive role in health prevention and risk management, providing essential data for regulatory agencies and policymakers. Recent research in Europe has highlighted the widespread presence of pesticide metabolites in both urban and rural populations, with particularly high exposure levels observed in children, considered a vulnerable group. In Slovenia, 74 urinary biomarkers, including banned pesticides and emerging substances, have been identified in children, revealing the presence of multiple environmental chemicals during early childhood. Some studies have reported associations between prenatal pesticide exposure, as measured in maternal and umbilical cord blood, and adverse birth outcomes such as preterm birth and low birth weight, highlighting the need for further research to clarify causal relationships [148,149].

Studies conducted in New Zealand and Ethiopia have identified varying patterns of pesticide exposure, assessed via urinary metabolites, influenced by seasonal factors, dietary habits, and occupational activities. Notably, cases of pesticide poisoning were documented among workers in floriculture enterprises [150,151]. Such geographic differences in exposure emphasize the need to consider both climate variability and trade-driven pesticide distribution when interpreting biomonitoring data.

Emerging evidence also indicates possible involvement of pesticides in molecular and epigenetic alterations. The *Parental Pesticide and Offspring Epigenome* (PaPoE) study, conducted in Morocco, linked parental pesticide exposure, measured in blood, to altered DNA methylation profiles in cord blood of offspring, suggesting potential intergenerational effects. Similarly, another study documented oxidative DNA damage in Cypriot children, assessed through urinary levels of glyphosate metabolites and 8-hydroxy-2′-deoxyguanosine (8-OHdG), reinforcing concerns about the genotoxic potential of currently used pesticide residues [152,153].

Large-scale projects such as *Human Biomonitoring for Europe* (HBM4EU) [154,155,156] and the *European Partnership for the Assessment of Risks from Chemicals* (PARC) [157] are significantly contributing to the development of reference databases on human exposure, useful for guiding health and environmental policies.

Despite significant progress in recent years, current systems for monitoring pesticide exposure remain largely focused on residues in agricultural products and food, failing to capture the complexity of real-world exposure. This includes non-dietary routes such as inhalation or dermal absorption, exposure to heterogeneous chemical mixtures, and impacts on particularly vulnerable life stages such as childhood and pregnancy [148,149].

To more effectively address these challenges, a structural shift is needed to systematically include HBM in both national and European surveillance programs. This approach should be complemented by integrated environmental monitoring, capable of detecting the persistence and bioaccumulation potential of certain pesticides, which may remain in ecosystems and food chains long after application. Strengthening the link between environmental indicators and public health data, through tools such as ecotoxicological biomarkers or sentinel species, would enable a more realistic, preventive, and evidence-based approach to pesticide regulation. Integrating HBM findings with trade and climate data will allow for more targeted policies that account for the dynamic environmental and economic factors affecting human exposure.

However, critical knowledge gaps remain, particularly regarding long-term health effects and the translation of biomonitoring data into risk management policies. Methodological inconsistencies persist, along with limited geographic coverage and poor representativeness of the most exposed populations. There is an urgent need to develop standardized methodologies, promote studies in low- and middle-income countries by strengthening infrastructure, and integrate biomonitoring with participatory surveillance systems and a clear focus on environmental justice. This requires greater inclusion of socially and geographically marginalized groups, enhanced stakeholder engagement, and effective risk communication.

Reconceptualizing HBM not only as a scientific endeavor but also as a socio-political tool will allow the field to contribute more meaningfully to the protection of environmental health on a global scale.

### 5.2. Alternative Models and Advanced Technologies

A radical shift is currently taking place in the scientific tools available for pesticide risk assessment, with the aim of eliminating approaches that rely on animal testing. Among the alternatives to in vivo experimentation, in silico methods are demonstrating great potential, driven by recent advances in computational resources and the application of artificial intelligence and machine learning to large, curated datasets [158,159,160].

One widely used in silico approach is the *Quantitative Structure–Activity Relationship* (QSAR), a modeling technique applied in chemistry, pharmacology, and toxicology to predict the biological activity, properties, or behavior of chemical compounds based on their molecular structure. This method can be effectively integrated with machine learning techniques. Recent studies have confirmed the reliability and utility of in silico models in predicting acute toxicity across different chemical domains. The Leadscope QSAR model was evaluated for predicting the *United Nations Globally Harmonized System* (GHS) hazard categories, using acute oral toxicity data in rats. The study included pharmaceuticals, plant protection products, pharmaceutical intermediates, and their metabolites, and demonstrated the high reliability of the model in forecasting risks across a wide variety of chemical structures, making it applicable to several industrial sectors. The findings also emphasized the importance of a weight-of-evidence approach, which increases the accuracy and appropriateness of predictive modeling [161].

Although many studies have explored acute toxicity prediction in different species through in silico methods, most of them have focused on non-human endpoints, such as LC_50_ or LD_50_ values in rodents, fish, or birds. Decision tree-based QSAR models have been applied to evaluate the toxicity of pesticides in various bird species, addressing regulatory needs related to ecological protection rather than human health. Acute toxicity syndromes in fish have also been identified through a QSAR approach, which proved useful for mechanistic categorization but revealed limitations due to species specificity. More recently, chemometric approaches have been used to predict pesticide toxicity in chickens, reflecting the increasing refinement of computational models in the field of ecotoxicology [162,163,164,165].

Nonetheless, despite these advancements, a significant research gap remains concerning the development of QSAR or hybrid models capable of predicting human-specific acute toxicity endpoints, such as the *minimum toxic dose* (or *Toxic Dose Low*, TD_Lo_) [166]. This underlines the urgent need for a solid and interpretable computational framework that utilizes available chemical and biological data to anticipate acute toxicity risks to human health. The current study [166] addresses this unmet need by developing the *Quantitative Read Across Structure–Toxicity Relationship* (q-RASTR) model, which merges the strengths of traditional QSTR methods with the read-across technique, in compliance with OECD principles and relevant Testing Guidelines (TG 440, 443, 456, 458) [116,117,118,119].

*Physiologically Based Pharmacokinetic* (PBPK) models provide a detailed simulation of how chemical compounds are distributed and metabolized within the body, using realistic representations of organs and tissues. These models consist of interconnected sub-models for each physiological compartment, linked by blood flow and parameterized using known anatomical and physiological data. Unlike classical compartmental models, which rely on empirical volumes and constants, PBPK models are grounded in measurable biological processes, making them more realistic and predictive in describing chemical kinetics [167].

PBPK models can be developed using a bottom-up approach, based on in vitro studies, the literature, or known physicochemical properties, or through a top-down approach, which involves adjusting parameters based on in vivo experimental data. In the case of pyrethroids, permethrin has been used as a model compound to validate predictive tools for human exposure, including a PBPK model and the high-resolution *Stochastic Human Exposure and Dose Simulation* (SHEDS) model, taking advantage of the extensive exposure and toxicokinetic data available for this substance [168,169,170].

Nevertheless, one of the most critical issues is species extrapolation, since scaling results from animal models to humans introduces uncertainties that must be carefully addressed. These uncertainties are typically managed through the application of guidance documents and standardized methodologies that incorporate uncertainty factors, PBPK modeling, and interspecies scaling approaches. Additional concerns include inter-individual variability, the limited availability of high-quality in vivo data for parameterization, and the lack of harmonized validation criteria across regulatory bodies.

Reference documents now exist that address many of the barriers to regulatory adoption of PBPK models. The *International Programme on Chemical Safety*/*World Health Organization* (IPCS/WHO) Harmonization Report [171,172] and the U.S. EPA Guidance [173] define principles of good practice for model development, emphasizing transparency, documentation of assumptions and parameter sources, and the systematic use of sensitivity and uncertainty analyses. Both publications recommend comparing model predictions with independent experimental data as a key element of validation. The OECD Guidance [174] translates these principles into operational tools for regulatory use, introducing minimum reporting standards, templates and checklists, both qualitative and quantitative validation criteria, as well as recommendations for peer review and traceability of input data; together, these elements facilitate harmonization of assessments across jurisdictions and contribute to more consistent acceptance decisions.

In parallel, the study by McLanahan et al. [175] draws attention to essential practical aspects for regulatory adoption. In particular, the importance of code and dataset accessibility, independent validation, and transparent communication of uncertainty is emphasized. The authors call for closer collaboration between academia, industry, and regulatory authorities to ensure reproducible and fit-for-purpose models. These guidelines address typical critical issues in a complementary manner by defining procedures and templates for validating and reporting predictive performance. Furthermore, they require that interspecies scaling choices be made explicit and justified, with the possibility of parameterization through in vitro and in silico approaches and with assessment of the related uncertainties. Another element is the inclusion of population variability, such as population PBPK or Monte Carlo simulations, and the support for the use of read-across and alternative approaches to address the paucity of in vivo data, always accompanied by sensitivity and uncertainty analyses. Consistent adoption of these requirements, clear declaration of intended use, complete documentation of the structure and parameters, sensitivity and uncertainty analyses, comparison with independent data, and transparent sharing of supporting material, substantively strengthens the evidentiary basis for the use of *Physiologically Based Pharmacokinetic*/*Physiologically Based Toxicokinetic* (PBPK/PBTK) models in regulatory decisions.

Another interesting case involves prothioconazole (PTC), a triazole fungicide that has shown strong in vitro inhibition of important cytochrome P450 (CYP450) enzymes involved in drug metabolism. To assess the risk of in vivo inhibition, a *Physiologically Based Toxicokinetic* (PBTK) model, conceptually equivalent to a PBPK model but applied in a toxicological context, was used. Building a PBTK model for PTC has been challenging due to the limited availability of human pharmacokinetic data, often restricted for ethical reasons. Nevertheless, adherence to PBTK/PBPK modeling principles consistent with international guidance allowed to explicitly evaluate the population variability and to support the conclusion of absence of clinically relevant *Pesticide–Drug Interactions* (PDI) at exposure levels close to the *Acceptable Daily Intake* (ADI), illustrating how adherence to the guidelines improves the robustness and acceptability of the assessments. Furthermore, the model allowed us to assess interindividual variability in PDI, based on the different metabolic phenotypes of the cytochrome P450 2C9 (CYP2C9) enzyme present in the population. Overall, the results support the reliability of the current regulatory exposure limits and confirm the value of the PBTK and PBPK models as robust and predictive tools for assessing health risks associated with pesticide exposure [176,177].

At the same time, omics technologies, such as genomics, transcriptomics, proteomics, metabolomics, and epigenomics, are increasingly employed to detect early, sub-lethal, or systemic biological responses to pesticide exposure. These approaches provide a detailed and multidimensional analysis of molecular changes induced by environmental contaminants. Recent research has shown that multi-omics integration is an effective strategy for overcoming the limitations of individual technologies, enabling a more comprehensive and unified understanding of pesticide mechanisms of action at both the cellular and systemic levels.

For example, genomics and transcriptomics have been used to identify genes regulated by pesticides like dichlorodiphenyltrichloroethane (DDT) and chlorpyrifos, which are involved in neuronal apoptosis as well as cardiac and neural development. Proteomic analyses have revealed significant changes in protein expression in model organisms such as zebrafish and plaice exposed to insecticides, including alterations in key proteins like *Heat Shock Protein* 70 (HSP70), CYP450, and metallothioneins. Metabolomic techniques, using liquid chromatography coupled with mass spectrometry (LC-MS) and gas chromatography coupled with mass spectrometry (GC-MS), have shown profound shifts in metabolic profiles even at low exposure doses, pointing to disruptions in energy metabolism, changes in gut microbiota composition, and potential links to disorders such as autism spectrum conditions. Epigenomic studies have further demonstrated that pesticide exposure can induce epigenetic modifications, including DNA methylation, histone changes, and alterations in microRNA, potentially leading to transgenerational effects, as observed in research on prenatal glyphosate exposure [138,178,179].

Together, these omics technologies offer a powerful toolset for understanding the mechanisms of pesticide toxicity, identifying predictive biomarkers, and integrating environmental and health risk assessments in a more sophisticated manner.

In parallel, the *European Safe and Sustainable by Design* (SSbD) framework, outlined in the Chemicals Strategy for Sustainability of the EU, encourages the development of chemical substances that are not only effective but also inherently safer. The application of green chemistry principles and the *Design for Environment* (DfE) approach supports the creation of molecules that are more biodegradable, less persistent, and with reduced bioaccumulation potential [8,180,181,182]. Additionally, emerging technologies such as exposomics and blockchain-based traceability offer promising avenues to enhance pesticide governance. Exposomics allow for the comprehensive assessment of real-world human and environmental exposures to chemical mixtures, linking these exposures to early biomarkers of effect and improving cumulative risk assessment models [183,184,185]. Blockchain technologies, on the other hand, can increase transparency and traceability across the entire pesticide supply chain, from production to field application, ensuring compliance with regulatory standards and supporting data-driven decision-making [186,187]. The integration of these approaches with omics data and SSbD strategies could enable more proactive, precise, and resilient management of chemical risks, fostering both environmental protection and sustainable agricultural practices.

These strategies are essential to decouple agricultural innovation from ecological degradation. They contribute to building a more dynamic, ethical, and economically sustainable regulatory system, one that is better prepared to evaluate new active substances in future pesticide regulatory frameworks.

### 5.3. Regulatory Review and the Precautionary Principle

The evolution of the scientific landscape calls for an integrated and innovative approach, not only on the technological front but also from a regulatory perspective, to ensure effective and up-to-date management of chemical risks arising from pesticides and other substances. Foundational regulations such as Regulation (EC) No. 1107/2009 [5] and Regulation (EC) No. 1907/2006 (REACH) [6] remain essential pillars in chemical risk governance; however, they do not adequately address the complexities associated with cumulative exposure, the effects of chemical mixtures, and emerging endpoints such as endocrine disruption, reprotoxic compounds, immunotoxic substances and epigenetic toxicity, phenomena that are now widely recognized in the scientific literature.

The precautionary principle must therefore be explicitly and systematically integrated into assessment and authorization processes, with particular attention to substances suspected of having endocrine or reproductive toxicity. In this context, *Cumulative Risk Assessment* (CRA) is emerging as a paradigm that goes beyond the traditional single-substance approach. CRA, as developed and promoted by EFSA through *Cumulative Assessment Groups* (CAGs), seeks to evaluate the combined effects of substances that share similar toxicological modes of action or common target organs, enabling a more realistic estimation of population risk.

Human exposure to chemicals involves a complex mixture of compounds originating from various sources, with food consumption representing the primary route of intake for many pesticides and environmental contaminants. CRA incorporates individual dietary consumption data with chemical occurrence data from official monitoring programs—such as the *EU multi-annual control programmes* [188], using probabilistic methodologies to provide a more accurate and representative evaluation of real-world exposures. Key quantitative tools include the *Hazard Index* (HI), the *Relative Potency Factor* (RPF), and the *Toxic Equivalency Factor* (TEF), which allow for normalization of cumulative exposure in reference to a benchmark compound. These tools facilitate risk characterization and help identify potential exceedances of safety thresholds [189,190].

The tiered approach adopted in CRA enables an initial conservative assessment, followed, where necessary, by more refined and specific evaluations (Tier 2). This strategy optimizes resource use and improves the precision of risk estimation. Risk communication relies on the *Margin of Exposure Total* (MoET), with threshold values aligned with traditional toxicological safety margins (e.g., MoET ≥ 100 at the 99.9th percentile), which serve as guides for regulatory decision-making [191].

Although the EFSA CAG framework follows a structured methodology, a systematic quantitative evaluation of its statistical power and uncertainty is still lacking. While probabilistic approaches and RPF normalization facilitate risk characterization, the magnitude of uncertainty across chemicals, population subgroups, and model assumptions remain largely unquantified. Recognizing this limitation is essential, and further research is needed to validate assumptions, perform sensitivity analyses, and reinforce confidence in the framework for regulatory governance.

Despite methodological progress, significant challenges remain. These include the need to harmonize the regulatory framework between REACH and plant protection product legislation, reducing inconsistencies in classification, data requirements, and evaluation timelines to avoid inefficiencies and regulatory gaps. Furthermore, it is necessary to integrate the REACH regulation with workplace exposure provisions. The EU Chemicals Strategy for Sustainability proposes mechanisms for joint review of authorization procedures, promoting transparency, reducing duplication, and fostering a more coherent policy landscape.

It is also essential to incorporate *Human Biomonitoring* (HBM) data into cumulative risk assessments and to account for toxicodynamic interactions, which remain underexplored. Advanced tools such as the *Aggregate Exposure Pathway* (AEP) framework and integrated exposure and biokinetic modeling platforms, such as the U.S. EPA models *Exposure Forecasting* (ExpoCast) and SHEDS, offer promising perspectives for more comprehensive integration of multiple exposures into regulatory models [137,191,192].

## 6. Conclusions

Pesticide chemical risk assessment is at a decisive crossroads. Mounting evidence from human biomonitoring, mixture toxicology, and mechanistic studies reveals that current models, based largely on isolated substances and simplified exposure scenarios, are insufficient to safeguard human and environmental health.

The next generation of risk assessment must be multidimensional, anticipatory, and grounded in real-world conditions. This means embracing novel tools such as omics technologies, in vitro/in silico models, and human biomonitoring, alongside implementing green chemistry and SSbD principles. Crucially, regulatory frameworks must evolve in parallel to keep pace with scientific advancement and societal expectations.

The precautionary principle should remain central to decision-making, particularly when addressing emerging risks like endocrine disruptors, reprotoxic compounds, or substances with unclear cumulative effects, and immuno-toxic compounds. Enhanced data transparency, methodological harmonization, and regulatory adaptability are essential to reduce long-term risks, protect future generations, and support the ecological transition of European agriculture.

Delaying this transformation risks not only public and environmental health, but also the strategic competitiveness of the agricultural and agrochemical sectors of the EU. Only through an integrated, forward-looking and science-based approach can we achieve a sustainable balance between productivity, human well-being, and environmental resilience.

In conclusion, an effective chemical risk assessment and management system must combine scientific innovation, regulatory integration, and a rigorous application of the precautionary principle. This is vital for protecting public health and environmental sustainability in a context of increasing and complex exposure to chemical mixtures, while at the same time promoting solutions that concretely address the needs of the agricultural sector and support its economic and productive sustainability.

## Figures and Tables

**Table 1:** The substances are divided into three categories according to the *Endocrine Disruptor List*. **List I**—Substances identified as EDs that have completed the full evaluation process for endocrine disruption under EU legislation, including the Plant Protection Products Regulation (PPPR), the Biocidal Products Regulation (BPR), or REACH (Candidate and Authorization Lists); **List II**—Substances currently under assessment in an EU legislative process due to explicit concerns about potential endocrine-disrupting properties; **List III**—Substances considered, by the evaluating National Authority, to have endocrine-disrupting properties based on scientific evidence. Abbreviations: Biocidal Products Committee (BCP); Community rolling action plan (CoRAP).

Active Substance	Chemical Class (Type)	Health Effects	Environmental Effects	Status (Status Year)	Regulatory Field
List I					
Asulam	Carbamate (herbicide)	No	Yes	Legally adopted (2024)	PPPR
Benthiavalicarb	Carbamate (fungicide)	Yes	No	Legally adopted (2024) Concluded ED in EFSA opinion (2021)	PPPR
Clofentezine	Tetrazine (acaricide)	Yes	No	Legally adopted (2024) Concluded ED in EFSA opinion (2021)	PPPR
Cyanamide	Nitrile (herbicide)	Yes	Yes	Legally adopted (2024) Concluded ED in EFSA opinion (2021)	BPR
Dimethomorph	Morpholine (fungicide)	Yes	Yes	Legally adopted (2024) Concluded ED in EFSA opinion (2023)	PPPR
Mancozeb	Dithiocarbamate (fungicide)	Yes	Yes	Legally adopted (2021) Concluded ED in EFSA opinion (2020)	PPPR
Mepanipyrim	Aminopyrimidines (fungicide)	Yes	Yes	Legally adopted (2024) Concluded ED in EFSA opinion (2023)	PPPR
Metiram	Dithiocarbamate (fungicide)	Yes	No	Legally adopted (2024) Concluded ED in EFSA opinion (2023)	PPPR
Metribuzin	1,2,4-triazines (herbicide)	Yes	No	Legally adopted (2024) Concluded ED in EFSA opinion (2023)	PPPR
Propiconazole	Triazole (fungicide)	Yes	Yes	Legally adopted (2024) Concluded ED in BPC opinion (2022)	PPPR
Triflusulfuron-methyl	sulfonylurea (herbicide)	Yes	No	Legally adopted (2024) Concluded ED in EFSA opinion (2022)	PPPR
**List II**					
Deltamethrin	Pyrethroid (type 2) ester (insecticide)	Yes	No	Commission EDC list (2019)	Cosmetics
Ziram	Dimethyldithiocarbamate (fungicide)	Yes	Yes	CoRAP (ECHA) list (2012)	REACH
Diuron	Urea (herbicide)	Yes	Yes	Concluded ED in SEV (2024) CoRAP (ECHA) list (2012)	REACH
Thiabendazole	Benzimidazoles (fungicide)	Yes	No	Concluded ED in EFSA opinion (2022)	PPPR
Fludioxonil	Phenylpyrrole (fungicide)	Yes	Yes	Concluded ED in EFSA opinion (2024)	PPPR
Flufenacet	Thiadiazole (herbicide)	Yes	Yes	Concluded ED in EFSA opinion (2024)	PPPR
Cyprodinil	Anilinopyrimidine (fungicide)	Yes	Yes	Concluded ED in EFSA opinion (2025)	PPPR
3-iodo-2-propynylbutylcarbamate (IPBC)	Carbamate (fungicide)	No	Yes	Concluded ED in BPC opinion (2024)	BPR
**List III**					
Prochloraz	Imidazole (fungicide)	Yes	No	List III National Authority evaluation (2020)	-

## Data Availability

No new data were created or analyzed in this study.
